# Copy number variation detection using next generation sequencing read counts

**DOI:** 10.1186/1471-2105-15-109

**Published:** 2014-04-14

**Authors:** Heng Wang, Dan Nettleton, Kai Ying

**Affiliations:** 1 Lyman Briggs College, Michigan State University, East Lansing, USA; 2 Department of Statistics and Probability, Michigan State University, East Lansing, USA; 3 Department of Animal Science, Michigan State University, East Lansing, USA; 4 Department of Statistics, Iowa State University, Ames, USA; 5 Genome Technology Branch, The National Human Genome Research Institute, NIH, Bethesda, USA

**Keywords:** Count data, Gamma-Poisson mixture, Hidden Markov model, Plant genomics, Poisson mixture model

## Abstract

**Background:**

A copy number variation (CNV) is a difference between genotypes in the number of copies of a genomic region. Next generation sequencing (NGS) technologies provide sensitive and accurate tools for detecting genomic variations that include CNVs. However, statistical approaches for CNV identification using NGS are limited. We propose a new methodology for detecting CNVs using NGS data. This method (henceforth denoted by m-HMM) is based on a hidden Markov model with emission probabilities that are governed by mixture distributions. We use the Expectation-Maximization (EM) algorithm to estimate the parameters in the model.

**Results:**

A simulation study demonstrates that our proposed m-HMM approach has greater power for detecting copy number gains and losses relative to existing methods. Furthermore, application of our m-HMM to DNA sequencing data from the two maize inbred lines B73 and Mo17 to identify CNVs that may play a role in creating phenotypic differences between these inbred lines provides results concordant with previous array-based efforts to identify CNVs.

**Conclusions:**

The new m-HMM method is a powerful and practical approach for identifying CNVs from NGS data.

## Background

### Introduction

A copy number variation (CNV) is a variation between genomes in the number of copies of a genomic region that is 1,000 DNA bases (1 Kb) or larger [1]. A CNV is a type of structural variation (SV) because a CNV affects a relatively large region in a DNA molecule. Structural genomic duplications or deletions correspond to copy number gains or losses, respectively. CNVs play an important role in human hereditary illnesses [2] and in plant breeding and agricultural improvement [3].

Maize exhibits extensive variation in both genotype and phenotype relative to the variation seen in humans [4]. The genotypic diversity in maize species permits a variety of uses, such as human and animal food and fuel. The maize genotype B73 was sequenced in 2009 [3]. This accomplishment allows a further comparison and understanding in different types of maize. Swanson-Wagner et al. [5] and Belo et al. [6] compared a variety of maize inbreds with B73 using array comparative genomic hybridization (aCGH) and identified a considerable number of CNVs along the genome. Springer et al. [7] also analyzed the structural variance between the two maize genotypes B73 and Mo17 using aCGH.

Array comparative genomic hybridization, first proposed in 1997 [8], has served as a robust and effective approach for CNV screening [9]. Statistical methods for analyzing aCGH data are readily available and are described in review articles such as Wineinger et al. [10] and Medvedev et al. [11]. However, aCGH is expensive and has limited resolution and accuracy. Nowadays, rapidly developing next generation sequencing (NGS) technologies provide a sensitive and accurate alternative approach for accessing genomic variations. The quality, speed, and affordability give NGS a significant advantage over microarrays [12,13].

Despite the advantages of NGS over aCGH, the use of NGS for CNV identification has been limited by a lack of available and effective statistical approaches. The well-developed aCGH data analysis methods cannot take the full advantage of NGS data, and thus, new statistical analysis methods for NGS data are needed. Most of the existing methods for CNV detection using NGS data can be classified into two categories: sliding window methods and hidden Markov model (HMM) methods. Sliding window methods include Segseq by Chiang et al. [14], Event-wise testing by Yoon et al. [15], rSW-seq by Kim et al. [16] and JointSLM by Magi et al. [17], among others. This category of methods must simultaneously deal with a large number of tests of significance, and the results of such methods are highly dependent on the determination of critical values. One example of the methods using HMMs is CNAseg by Ivakhno et al. [18]. This method relies on borrowing information from additional samples of the two genomes in comparison. In most situations, only one sequenced sample from each genotype is available. The method proposed in this paper is able to detect copy number variation change points even with just one sequenced sample of each genotype.

### Data collection and terminology

To understand the data and our model for the data, it is necessary to introduce some NGS data collection details and terminology.

First of all, we use *reference genome* to describe the genome of the genotype that has been fully sequenced using whole-genome sequencing technologies. In contrast, the *target genome* is the genome of a genotype of interest that has not been fully sequenced. The goal is to use NGS data from the reference and target genotypes to identify regions of copy number variation between the reference and target genomes. We say that a genomic region in the target genotype where the number of copies is amplified relative to the reference genotype has a *copy number gain*. A target genomic region present, but at a reduced copy number relative to the reference genome, is said to have a *copy number loss*. A region that is present in the reference genome but absent in the target genome is described as *absent*. These three states (copy number gain, copy number loss, and absent) represent *copy number variations* in the target genome relative to the reference. A region with no difference in the number of copies between the target and the reference genotypes is said to be *normal* in state. A genomic location where there is a change from one copy number state to another is called a copy number *change point*.

To identify copy number change points and copy number states, a DNA sample from each of the target and the reference genotypes is obtained. The DNA strands in a sample are fragmented into 100 to 1,000 base segments. At one end of every randomly sampled segment, a sequence of 100 to 300 bases is determined and recorded. Such a sequence of bases is called a *read*. Each of the reads is then aligned to the reference genome to determine its origin in the genome. The location of the first base of the read on the reference genome is recorded as the position of the read. The numbers of reads for the target genome and the reference genome are recorded as the *target read counts* and the *reference read counts*, respectively. If a location has a positive target read count or a positive reference read count, it is called a *site*. Thus, data from NGS technologies are small non-negative integer counts with associated site positions on the reference genome.

### Preliminary data processing

The data to be analyzed can be described as follows. Suppose the observed reference and target genome read counts are denoted by $o_{i}^{\left[r\right]}$ and $o_{i}^{\left[t\right]}$, respectively. The corresponding genomic positions are denoted by *h*_*i*_, *i* = 1,…,*I*. The goal of m-HMM is to find the segmentation $h_{1}\equiv h_{i_{0}}<h_{i_{1}}<h_{i_{2}}<\ldots <h_{I}\equiv h_{i_{J}}$ such that the copy number of the target genome changes between two consecutive segments, and remains the same within a same segment. Because the read counts take small non-negative integer values, including a large number of zeros, it is difficult to carry out accurate modeling and inference using the original data. Thus, it is more practical to work with sums of counts rather than the original individual counts.

A common way to aggregate the data is to define windows with a specific width and calculate the sum of target and reference read counts within each window, so as to obtain shorter series of larger target and reference read counts. Kim et al. [16] defined windows using a fixed number of read counts in the reference genome. Chiang et al. [14], Xie et al. [19] and Ivakhno et al. [18] defined windows using a fixed genomic distance. These methods have an underlying assumption that the sites within a window share the same copy number state. Such an assumption may be reasonable because a CNV is a somewhat rare type of genomic mutation, and the closer any two sites are located on the genome, the less likely there is a CNV change point between these sites. However there is also a problem in implementing these methods. Sites are randomly located along a genomic sequence, with a high density in some parts of the genome and a low density in other parts of the genome. Rigidly defining windows with a fixed number of read counts has the potential to put sites physically far away from each other into one window, which increases the risk of including copy number change points in a window. Rigidly defining windows with a fixed genomic distance can produce high variation in the number of sites and in read counts across windows. This can lead to decreased accuracy for identifying CNVs.

In this paper, we propose a new grouping method, which takes into consideration both the numbers of sites in windows, and distances among sites within and between windows. We propose to use K-means clustering on the physical site positions to define windows. This method is implemented on each chromosome separately. We first divide each chromosome into several big parts, by defining breakpoints that correspond to the largest distances between adjacent sites. In real data set, we suggest to divide each chromosome into 20 parts. Then we perform K-means clustering for each of the 20 parts, where *K* is chosen for any particular part as the number of reference genomic sites in the part divided by a constant number *C*. Thus *C* is on average the number of reference genome sites in each window after grouping. In this paper, *C* = 40 is used in the simulation study and real maize data analysis. Finally, we obtain the windows, where each window is defined by a collection of sites in one cluster. The number of windows *W* may be different from one chromosome to another. In the maize application we present later in the paper, the number of windows per chromosome ranges from 2707 to 5831. For window *w* = 1,…,*W*, let *g*_*w*_ denote the set of indices corresponding to sites in window *w*. Thus we obtain a new sequence of the target genome read counts

$$\begin{array}{c} u_{1}^{\left[t\right]},u_{2}^{\left[t\right]},\ldots ,u_{W}^{\left[t\right]}, \end{array}$$

and a new sequence of the reference genome read counts

$$\begin{array}{c} u_{1}^{\left[r\right]},u_{2}^{\left[r\right]},\ldots ,u_{W}^{\left[r\right]}, \end{array}$$

where the target and reference read counts for window *w* are the sum of the target read counts and the sum of the reference read counts within that window: $u_{w}^{\left[t\right]}=\sum_{i\in g_{w}}o_{i}^{\left[t\right]}$, $u_{w}^{\left[r\right]}=\sum_{i\in g_{w}}o_{i}^{\left[r\right]}$, *w* = 1,…,*W*. We use the median position of sites within a window as the location for that window: *ℓ*_*w*_ = median{*h*_*i*_,*i* ∈ *g*_*w*_}, *w* = 1,…,*W* and obtain a series of genomic locations

$$\begin{array}{c} \ell_{1},\ell_{2},\ldots ,\ell_{W}. \end{array}$$

By this method, the sites that are closest together are more likely to be grouped together in a window, which results in a more reasonable grouping than previously used approaches.

Once sites are grouped into windows, we assume that the hidden states remain the same within windows, and our m-HMM method is used to obtain a window-based segmentation. Next, an adjustment procedure is further performed on the window-based result to obtain more accurate copy number change points. We refer to the final segmentation result as the site-based segmentation result.

### Mixture-Hidden Markov Model (m-HMM) for a window based result

In this section, we describe the first step of the proposed mixture-hidden Markov model (m-HMM) that we use to estimate the window-based copy number change points along the genome. This CNV-detecting methodology is carried out separately on each chromosome.

HMM was described by Baum et al. [20-23]. A HMM is constructed by a bivariate random process {*S*_*w*_,***U***_*w*_}, *w* = 1,…,*W*. One component of this random process, {*S*_*w*_} (*w* = 1,…,*W*), is an unobserved Markov chain with finite states. {*S*_*w*_} (*w* = 1,…,*W*) has Markov property, which means that given the "current state" *S*_*w*_, the "future state" *S*_*w*+1_ and the "past state" *S*_*w*-1_ are independent, i.e., *P*(*S*_*w*+1_|*S*_1_,…,*S*_*w*_) = *P*(*S*_*w*+1_|*S*_*w*_), *w* = 1,…,*W*. The probability *a*_*kl*_ = *P*(*S*_*w*+1_|*S*_*w*_) is called the *transition probability*, which determines the probability of the state of *w* + 1 based on the state of *w*. In m-HMM, the unobserved copy number states of the windows along the chromosome are the hidden states. The copy number states take four values, where

1 = gain: copy number gain/amplification in the target relative to the reference,

2 = normal: no difference in copy number between the target and the reference,

3 = loss: region present in the target genome but at a reduced copy number relative to the reference,

4 = absent: region absent in the sample but present in the reference.

Another important component of a HMM is {***U***_*w*_} (*w* = 1,…,*W*), which is a sequence of observations for *w* = 1,…,*W*. Each hidden state generates an observation with specific probability, *P*(***U***_*w*_|*S*_*w*_), which is called the *emission probability*. In m-HMM, the observations are the target genome read count $u_{w}^{\left[t\right]}$ and the reference read count $u_{w}^{\left[r\right]}$ at each window *w*, i.e., ***U***_*w*_ with value $u_{w}={\left(u_{w}^{\left[t\right]},u_{w}^{\left[r\right]}\right)}^{\prime }$, *w* = 1,…,*W*. Detailed description of the m-HMM is presented in the following two subsections.

#### The transition probabilities

For the copy number states *k* and *l*, the transition probability *a*_*kl*_(*w*) (*k*,*l* = 1,…,4) is defined as the conditional probability of the next window *w*+1 taking copy number state *l*, given the copy number state *k* for the current window *w*. Motivated by Marioni et al. [24], we define the transition matrix which takes the relative positions of windows into consideration. As the distance between adjacent windows increases, the transition probability from state *k* to state *l* ≠ *k* increases and approaches to a positive constant. As the distance between adjacent windows decreases, the probability of a difference in copy number states between windows diminishes.

For each window *w*, the transition probability is defined as a function of *w* given by ***A***_*w*_ = [*a*_*kl*_(*w*)]_4×4_, where

(1) $$\begin{array}{ll} \;a_{\text{kl}}(w) & =P(S_{w+1}=l|S_{w}=k,\theta )\\ & =\left\{\begin{array}{ll} p_{\text{kl}}(1-e^{-\rho d_{w}})\; & l\neq k\\ 1-(\sum_{j\neq k}p_{\text{kj}})(1-e^{-\rho d_{w}})\; & l=k \end{array}\right), \end{array}$$

for *k*,*l* = 1,…,4 and *w* = 1,…,*W* - 1. Here the parameter *p*_*kl*_ ∈ (0,1) affects the transition probabilities from state *k* to state *l*, and has the constraint $\sum_{l\neq k}p_{\text{kl}}<1$, *k* = 1,…,4. Constant *d*_*w*_ denotes the physical distance on the genome between the location of window *w* and the location of window *w* + 1 (i.e., *ℓ*_*w*+1_ - *ℓ*_*w*_). The parameter *ρ* is a positive-valued parameter determining the effect of distance on the transition matrix. The distance effect diminishes as *ρ* approaches *∞*. The parameter vector ***θ*** represents all the parameters in the model, including *p*_*kl*_ (*k*,*l* = 1,…,4;*l* ≠ *k*), *ρ* and all the parameters in the emission distribution to be described in the following section.

#### The emission distributions

The emission distributions define emission probabilities, which are the conditional joint probabilities of reference and target read counts, given the copy number state of the window. Each window *w* has two observations, a reference read count $U_{w}^{\left[r\right]}=u_{w}^{\left[r\right]}$ and a target read count $U_{w}^{\left[t\right]}=u_{w}^{\left[t\right]}$. We model the reference read count as $U_{w}^{\left[r\right]}|\lambda_{w}^{\left[r\right]}∼\text{Poisson}\left(\lambda_{w}^{\left[r\right]}\right)$, where $\lambda_{w}^{\left[r\right]}$ follows a Gamma distribution with parameters *α* and *β*.

Conditional on the state of window *w*, one natural choice for the emission distribution of the target read count for window *w* is

(2) $$\begin{array}{c} U_{w}^{\left[t\right]}|(\lambda_{w}^{\left[r\right]},S_{w}=k)∼\text{Poisson}(K_{k}c_{0}\lambda_{w}^{\left[r\right]}), \end{array}$$

Here *K*_1_ = 2, *K*_2_ = 1, *K*_3_ = 0.5 and *K*_4_ = 0 are the CNV effects of the four copy number states "gain", "normal", "loss" and "absent", respectively. Parameter *c*_0_ is a normalization factor that accounts for any discrepancy between the total number of reference and target reads in normal regions. However in real data sets, extra variation and mis-alignments along the genomic sequence data are inevitable. For example, in a normal genomic segment with no difference in copy number between the target and the reference, there still exist some windows with normalized target and reference read count ratios significantly higher or lower than 1; within a segment of copy number gain (or loss), we also find windows that have normalized target and reference read count ratios significantly lower than 2 (or higher than 0.5). The original HMM introduced above will not only identify true copy number variation signals, but also the local variations caused by random error. Without additional modification, the method tends to show more state changes than those justified by true CNV signals.

The problem of identifying too many CNV change points is also pointed out by Ivakhno et al. [18]. To address this problem, Ivakhno et al.’s CNAseg employs a merging adjustment procedure on the outcomes of the original HMM segmentations using Pearson’s *χ*^2^ statistics. However, CNAseg segmentation depends heavily on the determination of the merging threshold. In the method we propose, instead of using (2) to model the target read count distribution, we use a Poisson mixture model for each of the four copy number states *k* = 1,2,3,4:

(3) $$\begin{array}{c} U_{w}^{\left[t\right]}|\left(\lambda_{w}^{\left[r\right]},S_{w}=k\right)∼\overset{4}{\underset{j=1}{\sum }}q_{\text{kj}}\text{Poisson}\left(v_{\text{kj}}c_{0}\lambda_{w}^{\left[r\right]}\right), \end{array}$$

where $Q={\left[q_{\text{kj}}\right]}_{4\times 4}=\left(\begin{array}{cccc} q_{11} & 1-q_{11} & 0 & 0\\ \frac{1-q_{22}}{2} & q_{22} & \frac{1-q_{22}}{2} & 0\\ 0 & \frac{1-q_{33}}{2} & q_{33} & \frac{1-q_{33}}{2}\\ 0 & 0 & 1-q_{44} & q_{44} \end{array}\right)$ with *q*_*kk*_ ∈ (0.5,1) for *k* = 1,…,4, and $V={\left[v_{\text{kj}}\right]}_{4\times 4}=\left(\begin{array}{cccc} 2 & v_{12} & 0 & 0\\ v_{21} & 1 & v_{23} & 0\\ 0 & v_{32} & 0.5 & v_{34}\\ 0 & 0 & v_{43} & 0 \end{array}\right)$ with *v*_12_,*v*_21_ ∈ (1,2), *v*_23_,*v*_32_ ∈ (0.5,1), *v*_34_,*v*_43_ ∈ (0,0.5). The diagonal elements *v*_*kk*_ (*k* = 1,…,4) in ***V*** denote the effects of the four copy number states: gain, normal, loss and absent, and have fixed constant values *v*_11_ = 2, *v*_22_ = 1, *v*_33_ = 0.5 and *v*_44_ = 0. The off-diagonal elements *v*_*kj*_ (*k*,*j* = 1,…,4;*k* ≠ *j*) account for the uncertainties due to random errors in the sequencing technology such as mis-alignments, and their values are estimated from the data set. More specifically, parameter *v*_12_ accounts for the uncertainty in the copy number gain state that allows observed copy numbers to vary between 1 and 2; parameters *v*_21_ and *v*_23_ accounts for uncertainty in the normal state that allows observed copy numbers to vary between 0.5 and 2; parameters *v*_32_ and *v*_34_ allow observed copy numbers to vary between 0 and 1 within the copy number loss state; and the parameter *v*_43_ allows observed copy numbers to vary between 0 and 0.5 when the state is the absent state. All other *v*_*kj*_’s are restricted to 0. As a result, matrix ***V*** is a tridiagonal matrix. The weight matrix ***Q*** is also a tridiagonal matrix with *q*_*kj*_ (*k*,*j* = 1,…,4) being the weights for the Poisson components in the mixture distribution. Parameters *q*_*kk*_ (*k* = 1,…,4) denote the Poisson component weights for the four copy number states and are restricted between 0.5 and 1. Accordingly, the off-diagonal *q*_*kj*_’s are less than 0.5. In this way, the effects of the four copy number states dominate the effects of random uncertainties.

Based on the model as specified so far, the joint distribution for the target and the reference read counts at window *w*, conditional on the hidden state for window *w* being *k*, is

(4) $$\begin{array}{l} P^{\left(*\right)}\left(U_{w}^{\left[t\right]}=u_{w}^{\left[t\right]},U_{w}^{\left[r\right]}=u_{w}^{\left[r\right]}|S_{w}=k,\theta \right)\\ \;=\underset{j}{\sum }q_{\text{kj}}\frac{\Gamma \left(u_{w}^{\left[t\right]}+u_{w}^{\left[r\right]}+\alpha \right){\left(v_{\text{kj}}c_{0}\right)}^{u_{w}^{\left[t\right]}}\beta^{\alpha }}{\Gamma (\alpha )u_{w}^{\left[r\right]}!u_{w}^{\left[t\right]}!{\left(v_{\text{kj}}c_{0}+1+\beta \right)}^{u_{w}^{\left[t\right]}+u_{w}^{\left[r\right]}+\alpha }} \end{array}$$

with $u_{w}^{\left[r\right]}\in \mathbb{Z}\;\setminus \;{\mathbb{Z}}^{-}$ and $u_{w}^{\left[t\right]}\in \mathbb{Z}\;\setminus \;{\mathbb{Z}}^{-}$. A detailed derivation for (4) is provided in Additional file 1: Appendix 1. Note that

$$P^{\left(*\right)}\left(u_{w}^{\left[s\right]}=0,u_{w}^{\left[c\right]}=0|S_{w}=k,\theta \right)=\underset{j}{\sum }q_{\text{kj}}{\left(\frac{\beta }{v_{\text{kj}}c_{0}+1+\beta }\right)}^{\alpha },$$

which is greater than 0. However, it is not possible for both the target and the reference read counts to be zero in the same interval, i.e., the joint distribution of $u_{w}^{\left[t\right]}$ and $u_{w}^{\left[r\right]}$ is truncated in the sense that

(5) $$\begin{array}{c} P\left(u_{w}^{\left[t\right]}=u_{w}^{\left[r\right]}=0|\theta \right)=0. \end{array}$$

Consequently, we multiply the *j*^th^ component of (4) by a constant $\frac{{\left(v_{\text{kj}}c_{0}\;+\;1\;+\;\beta \right)}^{\alpha }}{{\left(v_{\text{kj}}c_{0}\;+\;1\;+\;\beta \right)}^{\alpha }\;-\;\beta^{\alpha }}$ (*j* = 1,2,3,4), and obtain the joint probability that satisfies (5). For the target and reference reads at window *w* given *S*_*w*_ = *k*, the joint emission probability is

(6) $$\begin{array}{l} P(U_{w}=u_{w}|S_{w}=k,\theta )\\ \;=\;\underset{j}{\sum }q_{\text{kj}}\frac{{\left(v_{\text{kj}}c_{0}\right)}^{u_{w}^{\left[t\right]}}\beta^{\alpha }}{u_{w}^{\left[t\right]}!\;u_{w}^{\left[r\right]}!\;\Gamma (\alpha )}\frac{\Gamma \left(u_{w}^{\left[t\right]}+u_{w}^{\left[r\right]}+\alpha \right)}{{\left(v_{\text{kj}}c_{0}+1+\beta \right)}^{u_{w}^{\left[t\right]}+u_{w}^{\left[r\right]}}\;\left({\left(v_{\text{kj}}c_{0}+1+\;\beta \right)}^{\alpha }-\beta^{\alpha }\;\right)}, \end{array}$$

where uw=uw[t]uw[r]∈(ℤ∖ℤ-)×(ℤ∖ℤ-)∖00 and $U_{w}=\left(\begin{array}{c} U_{w}^{\left[t\right]}\\ U_{w}^{\left[r\right]} \end{array}\right)$.

### Parameter estimation using the EM algorithm

We want to find the MLE for the parameter ***θ*** = {***p***,*ρ*,*α*,*β*,*c*_0_,***q***,***v***}, where ***p*** and *ρ* are transition probability parameters, and *α*, *β*, *c*_0_, ***q*** = (*q*_11_,*q*_22_,*q*_33_,*q*_44_)^′^ and ***v*** = (*v*_12_,*v*_21_,*v*_23_,*v*_32_,*v*_34_,*v*_43_)^′^ are emission probability parameters. It is not easy to directly maximize the likelihood with respect to this 26-dimensional parameter, so we use the Expectation-Maximization (EM) algorithm to iteratively maximize the likelihood

(7) $$L(\theta |u)=\;\underset{s}{\sum }\pi_{s_{1}}\overset{W-1}{\underset{w=1}{\prod }}a_{s_{w}s_{w+1}}(w)\overset{W}{\underset{w=1}{\prod }}P(u_{w}|s,\alpha ,\beta ,c_{0},q,v).$$

In (7), ***u*** = (***u***1′,…,***u****W*′)^′^ is a series of the target and reference read counts for all the windows along the specific chromosome, ***s*** = (*s*_1_,…,*s*_*W*_)^′^ ∈ {1,2,3,4}^*W*^ is a vector of unobserved states for all the windows along the chromosome, and $\pi_{s_{1}}=P(S_{1}=s_{1})$ is the probability that the copy number state in the first window is *s*_1_.

#### Characterizing the E and M steps

In the EM algorithm, the likelihood function is also called the observed data likelihood $L_{\text{(obs)}}=L(\theta |u)$ because all the observations ***u*** are observed. If the hidden states were known, we have the complete data likelihood function as follows:

(8) $$\begin{array}{l} L_{\text{(comp)}}=L(\theta |u,s)=\pi_{s_{1}}\overset{W-1}{\underset{w=1}{\prod }}a_{s_{w}s_{w+1}}\;(w)\overset{W}{\underset{w=1}{\prod }}\;P(u_{w}|s,\alpha ,\beta ,c_{0},q,v). \end{array}$$

In these two likelihoods, $\pi_{s_{1}}\prod_{w=1}^{W-1}a_{s_{w}s_{w+1}}(w)$ is the probability of that the hidden states for all the windows on the chromosome are *s*_1_,…,*s*_*W*_, respectively, and $\prod_{w=1}^{W}P(u_{w}|s,\alpha ,\beta ,c_{0},q,v)$ is the probability of the observed read counts ***u***, given the hidden states *s*_1_,…,*s*_*W*_.

Given the parameter estimates ***θ***^(*m*)^ from the iteration *m* of the EM algorithm, we use the E-step and the M-step to update the parameter estimate. 

• The E-step: Evaluate the expectation of the complete data log-likelihood with respect to the conditional distribution of the hidden states ***S*** given the observed data ***u***, with ***θ*** = ***θ***^(*m*)^, i.e., evaluate $E_{S|u,\theta^{\left(m\right)}}\left(\log L(\theta |u,s)\right)$.

• The M-step: Find ***θ***^(*m*+1)^, the ***θ*** value that maximizes $E_{S|u,\theta^{\left(m\right)}}\left(\log L(\theta |u,s)\right)$.

A detailed look at both the E and M steps is provided in the Additional file 1.

#### Initialization, convergence, and prediction of hidden states

The initial values for all the parameters are defined as follows. In the transition probabilities, we define the initial values $p^{\left(0\right)}\overset{\Delta }{=}{\left(p_{12}^{\left(0\right)},\ldots ,p_{43}^{\left(0\right)}\right)}^{\prime }=0.1\cdot 1_{12\times 1}$ and *ρ*^(0)^ = 0.5. For the parameters in the emission probabilities, we define $q_{11}^{\left(0\right)}=q_{22}^{\left(0\right)}=q_{33}^{\left(0\right)}=q_{44}^{\left(0\right)}=0.5$, $v_{12}^{\left(0\right)}=v_{21}^{\left(0\right)}=1.5$, $v_{23}^{\left(0\right)}=v_{32}^{\left(0\right)}=0.75$, $v_{34}^{\left(0\right)}=v_{43}^{\left(0\right)}=0.25$, and use the maximum likelihood estimates of *α* and *β* with all sites assigned with normal copy number state (state 2) as the initial values *α*^(0)^ and *β*^(0)^.

We use

$$x^{\left[t\right]}=\underset{w}{\sum }u_{w}^{\left[t\right]}I_{\left\{\text{window}\;w\;\text{with normal copy number state}\right\}}$$

and

$$x^{\left[r\right]}=\underset{w}{\sum }u_{w}^{\left[r\right]}I_{\left\{\text{window}\;w\;\text{with normal copy number state}\right\}}$$

to denote the total target and reference aligned counts for sites in normal copy number regions, and $c_{0}=\frac{x^{\left[t\right]}}{x^{\left[r\right]}}$ to denote the ratio between them. We obtain the initial estimate for *x*^[*t*]^ and *x*^[*r*]^ as follows. First we calculate the ratio between the read counts of the target genome and the reference genome for all the windows with positive reference genome counts through the whole genome across all chromosomes. Then we separate the ratios into three groups using K-means clustering to get three group means: *M*_1_, *M*_2_ and *M*_3_. Suppose *M*_1_ < *M*_2_ < *M*_3_. Then *x*^[*t*](0)^ is the sum of the target read counts for the windows belonging to the cluster with mean *M*_2_, and *x*^[*r*](0)^ is the corresponding sum of reference read counts for the windows belonging to the cluster with mean *M*_2_. After obtaining *x*^[*t*](0)^ and *x*^[*r*](0)^, we calculate $c_{0}^{\left(0\right)}$ using $\frac{x^{\left[t](0\right)}}{x^{\left[r](0\right)}}$.

The probabilities of the four hidden states for the first window $\pi_{s_{1}}$ (*s*_1_ = 1,2,3,4 and $\sum_{s_{1}=1}^{4}\pi_{s_{1}}=1$) are determined by the target and the reference read counts in the first window. We calculate

$$\begin{array}{c} R_{1}\overset{\Delta }{=}\frac{u_{1}^{\left[t\right]}}{u_{1}^{\left[r\right]}}\cdot \frac{x^{\left[r](0\right)}}{x^{\left[t](0\right)}}. \end{array}$$

If *R*_1_ > 1.5, then *π*_1_ = 1, i.e., we assign copy number gain as the initial copy number state to the first window; if 0.75 < *R*_1_ ≤ 1.5, then *π*_2_ = 1, i.e., we assign normal as the initial copy number state to the first window; if 0 < *R*_1_ ≤ 0.75, then *π*_3_ = 1, i.e., we assign copy number loss as the initial state to the first window; if *R*_1_ = 0, then *π*_4_ = 1, i.e., we assign absent as the initial state to the first window.

For all the other windows *S*_*w*_ (*w* ≥ 2), we use normal state (state 2) as the initial state. All the parameters are updated using the EM algorithm. The iteration stops when the difference between ***θ***^(*m*)^ and ***θ***^(*m*+1)^ is small enough, and we use the value of ***θ*** at the final iteration (denoted by $\hat{\theta }$) as our estimate of ***θ***.

After convergence, the conditional probability of each of the four states $P_{k}(w)=P\left(S_{w}=k|u,\hat{\theta }\right)$ (*k* = 1,2,3,4) is calculated, and the conditional hidden state prediction for window *w* is given by the state that has the largest conditional probability, i.e., ${Ŝ}_{w}=\underset{k}{\text{arg max}}\;P_{k}(w)$ for *w* = 1,…,*W*.

### Change point adjustment

**Figure 1:**
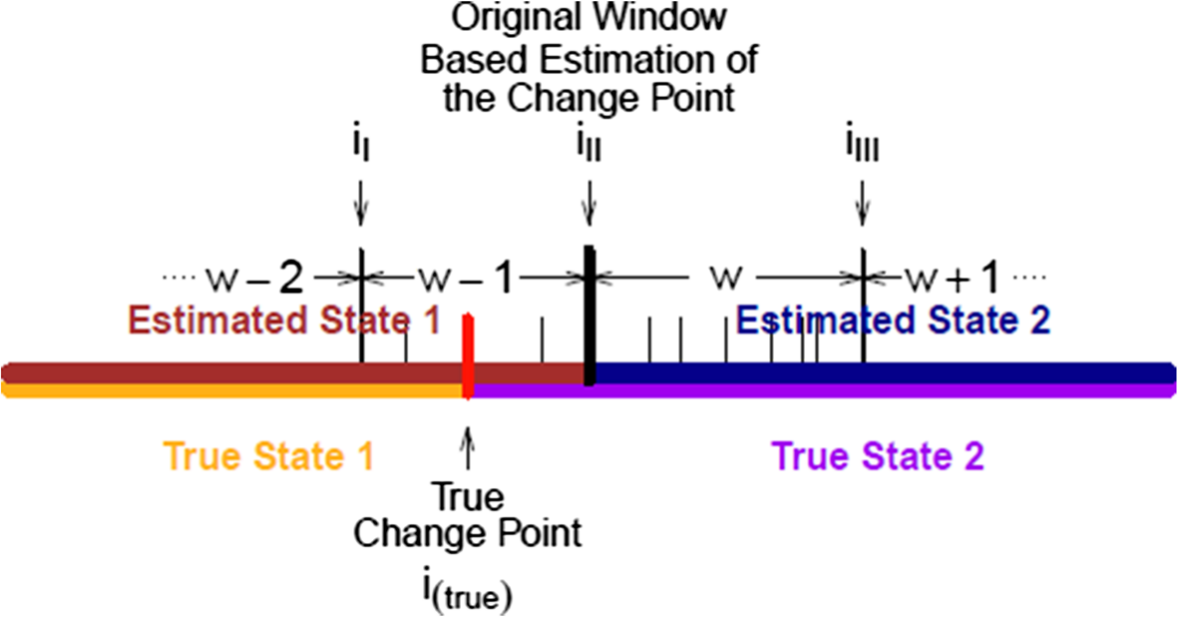
True change point and the estimate of the window based change point.

Sites are grouped into windows initially, because we wish to have larger read counts to better capture the copy number variation signals with the m-HMM, and it was assumed in the first step that the copy number state remains the same within a window. In reality, it is possible that the copy number change points occur within windows. Figure 1 demonstrates the relation between the estimated change point before adjustment and the true change point. Suppose window *w* is the window identified by the algorithm where the copy number state changes from state 1 to state 2 with genomic sites grouped into windows. In this plot, the brown color and the blue color segments represent the two sides of the window based segmentation cut point. And the vertical black bar is the boundary of the two windows (window *w*-1 and window *w*). Suppose *g*_*w*_ = {*i*_*II*_,*i*_*II*_ + 1,…,*i*_*III*_ - 1}, so *i*_*II*_ is the index of the first site in window *w*, which would be identified as the change point if no adjustment is made. With *g*_*w*-1_ = {*i*_*I*_,*i*_*I*_ + 1,…,*i*_*II*_ - 1} and *g*_*w*+1_ = {*i*_*III*_,*i*_*III*_ + 1,…,*i*_*IV*_-1}, we have *i*_*I*_ and *i*_*III*_ being the first sites of window *w* - 1 and window *w* + 1, respectively. The true change point *i*_(*true*)_ may happen between sites *i*_*I*_ and *i*_*III*_-1. In this plot, the true change point, represented by the vertical red bar, is within window *w*-1. In order to obtain a more accurate result, the following algorithm makes the adjustment: 

1. For site *i* between site *i*_*I*_ and site *i*_*III*_-1, we obtain both the total target and the reference counts from *i*_*I*_ to *i* - 1, denoted as $z_{i(L)}^{\left[t\right]}$ and $z_{i(L)}^{\left[r\right]}$; also obtain both the total target and the reference counts from *i* to *i*_*III*_ - 1, denoted as $z_{i(R)}^{\left[t\right]}$ and $z_{i(R)}^{\left[r\right]}$.

2. Calculate the Pearson’s *χ*^2^ test statistic $\sum_{\tau }\sum_{\gamma }\frac{{\left(z_{i(\gamma )}^{\left[\tau \right]}-{\hat{z}}_{i(\gamma )}^{\left[\tau \right]}\right)}^{2}}{{\hat{z}}_{i(\gamma )}^{\left[\tau \right]}}$ using $\left\{z_{i(L)}^{\left[t\right]},z_{i(L)}^{\left[r\right]},z_{i(R)}^{\left[t\right]},z_{i(R)}^{\left[r\right]}\right\}$, where *τ* = *t* or *r*, *γ* = *L* or *R*, and ${\hat{z}}_{i(\gamma )}^{\left[\tau \right]}=\frac{\left(\sum_{\gamma }z_{i(\gamma )}^{\left[\tau \right]}\right)\left(\sum_{\tau }z_{i(\gamma )}^{\left[\tau \right]}\right)}{\sum_{\gamma }\sum_{\tau }z_{i(\gamma )}^{\left[\tau \right]}}$.

3. Do step 1 and 2 for every site *i* between *i*_*I*_ and *i*_*III*_ - 1; the adjusted breakpoint *i*_(*adj*)_ is the one with the largest Pearson’s *χ*^2^ test statistic value.

## Results and discussions

### Simulation studies

To set up golden standard test data sets with known CNV position to evaluate the performance of our method and peer tools, we conducted three simulation studies. In the first and the second studies, we simulated reads counts on each of the target and reference positions, then we simulated CNV segments on the target genome. The simulated sequences in the first two studies are low coverage with depths of 2*X*. In the third simulation study, we first randomly simulated copy number variation segments, then we simulated pair end Illumina reads and mapped back to the reference genome. The third simulation captures the sequencing process and with high coverage with depths of 30*X*.

#### Simulation study 1

The first simulation study is based on real DNA sequencing data from chromosome 4 of the lung cancer cell line NCI-H2347 from Chiang et al. [14]. Simulation based on real data can best maintain the characteristic of the data including variation and errors that can affect actual data analyses. We first randomly simulated the genomic positions of the sites along the target and reference genomes using a uniform distribution. We generated the reference and target genome read counts by shuffling the reference genome read counts in chromosome 4 of NCI-H2347. After that, we randomly picked 90 CNV segments on the simulated target genome. We considered three sizes for the CNV segments: 10 kb, 50 kb and 100 kb and generated 10 segments for each CNV type. We doubled the read counts for the segments with copy number gains, halved the read counts for the segments with copy number losses and set read counts to 0 for segments with no copies. This is a low coverage sequencing with depth of 2*X*. We compare the result of the m-HMM with the mixture Poisson emission probability in (6), the result using the original HMM with the Poisson emission probability in (2), and the result using Segseq by Chiang et al. [14].

Table 1 is the comparison between the original HMM and the proposed m-HMM, as well as the m-HMM without change point adjustment. The four true copy number states are listed in the first column. Sensitivities, specificities, empirical false positive rate (EFPR) and empirical false negative rate (EFNR) are computed using (9) and listed in Table 1. From (9) we see that a more accurate method has greater values in sensitivity and specificity, and lower values in EFPR and EFNR.

(9) $$\begin{array}{l} \text{Sensitivity}=\frac{\#\text{correctly identified copy number changes of a given type}}{\#\text{true copy number changes of the given type}},\\ \text{Specificity}=\frac{\#\text{correctly identified normal sites}}{\#\text{true normal sites}},\\ \;\text{EFPR}=\frac{\#\text{incorrectly identified copy number changes of a given type}}{\#\text{identified copy number changes of the given type}},\\ \;\text{EFNR}=\frac{\#\text{incorrectly identified normal sites}}{\#\text{identified normal sites}} \end{array}$$

**Table 1:** Comparison among m-HMM with and without change point adjustment, and original HMM in simulation study 1

		**m-HMM**	**m-HMM no adj.**	**original HMM**
Gain	Sensitivity	0.917	0.915	0.863
	EFNR	0.097	0.174	0.751
Normal	Specificity	0.996	0.994	0.945
	EFPR	0.002	0.002	0.003
Loss	Sensitivity	0.858	0.831	0.826
	EFNR	0.309	0.434	0.846
Absent	Sensitivity	0.960	0.857	0.810
	EFNR	0.011	0.005	0.000

Sensitivities and empirical false negative rates for copy number gain, loss and absent states, as well as specificities and empirical false positive rates for the normal state in the first simulation study.

From Table 1, both the m-HMM with and without change point adjustment have greater sensitivities and specificity in all four copy number states than the original HMM. Also, the two m-HMM methods have lower EFPR and EFNR in copy number gain, loss and normal states. The original HMM has a slightly smaller EFNR for the absent state, but the EPNRs in copy number gains and losses are far greater than other methods. This means that, with the mixture Poisson emission distribution, the m-HMM is less affected by the errant variations in states and can capture true CNV signals better.

Comparing between the m-HMM with and without change point adjustment, the sensitivities and specificity are all increased by using the adjustment. Also, the EFPR and EFNR are decreased in copy number gain, loss and normal states, while increased slightly in the absent state. In general, the change point adjustment procedure does give the m-HMM better accuracies.

Table 2 lists a comparison between the m-HMM and SegSeq. Segseq classifies the copy number states with three categories: normal, copy number gain, and copy number loss. Thus, Segseq does not distinguish between the copy number loss state and the no copy state. In Table 2, Segseq did not identify any sites with copy number gain state in this simulation study, so both the sensitivity and EFNR are 0 in the copy number gain state. Moreover, SegSeq has a large EFNR and a low sensitivity in the loss or absent state. Although SegSeq was slightly better in identifying normal states, this came with the cost of much poorer identification of gains, losses, and absent sites by SegSeq relative to the m-HMM method.

**Table 2:** Comparison between m-HMM and SegSeq in simulation study 1

		**m-HMM**	**SegSeq**
Gain	Sensitivity	0.917	0.000
	EFNR	0.097	0.000
Normal	Specificity	0.996	0.998
	EFPR	0.002	0.019
Loss or absent	Sensitivity	0.898	0.076
	EFNR	0.223	0.678

Sensitivities and empirical false negatives rates for copy number gain, loss and absent states, as well as d positive rates for the normal state in the first simulation study.

#### Simulation study 2

In this simulation study, we examined the m-HMM for copy number variation segments with different lengths. We simulated 30 copy number variation segments with each of the following lengths: 10, 20, 30, 50 and 100 kb. Within the 30 copy number variation segments of the same length, we have 10 segments with copy number gain, 10 segments with copy number loss and 10 segments absent in the sample but present in the reference. The simulation procedure is the same as Simulation Study 1. An identification is considered successful if there is a non-empty intersection between an identified segment and a simulated copy number variation segment with the correct variation type. The numbers of successful identified segments out of 10 are listed in Table 3. When the CNV segments are 30 kb or longer the m-HMM method identifies almost all the copy number variation segments of any variation type.

**Table 3:** m-HMM segmentation with different CNV lengths in simulation study 2

**Lengths**	**10 kb**	**20 kb**	**30 kb**	**50 kb**	**100 kb**
Loss	0	4	9	10	10
Gain	3	5	10	10	10
Absent	2	9	10	10	10

#### Simulation study 3

In order to examine m-HMM methodology with a simulation data set that captures the sequencing process, we conducted a third simulation study. First, we randomly introduced duplications (from 3 copies to 4 copies) and deletions (either homozygous 0 copy or heterozygous 1 copy) with difference sizes (1 kb, 5 kb, 10 kb) in the maize reference genome version 2 (chromosome 6 from website ftp://ftp.ensemblgenomes.org/pub/plants/release-10/fasta/zea_mays/dna/) to produce a new genome with CNVs. Five segments were simulated for each of the 20 different combinations (4 different copies × 3 sizes). Consequently, we simulated 10 segments with copy number gains, and 10 segments with copy number losses or absence with each of the 3 CNV segment sizes. After that, we simulated randomly sampled paired end Illumina reads based on the new genome sequence with both read ends of length 100 bp, clone length of average 500 bp and standard deviation of 50 bp, coverage depth of 30*X* and sequence error rate of 0.002. Then, the simulated sequence reads are mapped back to reference genome using the same parameter as before to produce our test data set with known CNV copy number and position.

We compared the m-HMM with SegSeq and CNVnator by Abyzov et al. [25]. The simulation results are listed in Table 4 and Table 5. Table 4 lists the number of correctly detected segments for copy number gain, loss and absent states in the first two rows, and the number of incorrectly detected segments for the true normal state in the last row. Because neither SegSeq nor CNVnator distinguish between copy number losses and absence, the table summarizes the two deletion states in one row. We have 10 segments of copy number gain or loss/absent for each of the three sizes. When the CNV lengths are at least 5 kb, the m-HMM identifies all the CNV segments. CNVnator detected all the segments with losses or absent state, and almost all segments with copy number gains. SegSeq detected all the segments with copy number gains, but only detected about half of the loss/absent segments. When segment length equals 1 kb, the m-HMM identifies all but one segment, while the detection accuracies for SegSeq and CNVnator are much decreased. The last row of Table 4 lists the number of true normal segments that are incorrectly detected by the methods. Smaller numbers in this row represent better performance. Our proposed m-HMM has 3 incorrectly detected segments, which is fewer relative to the other two methods. CNVnator has a large number of false positive detections when the true copy number state is normal.

**Table 4:** Counts of correctly detected CNV segments and false positively detected normal segments, comparing between m-HMM, SegSeq and CNVnator in simulation study 3

	**Detected**	**m-HMM**			**SegSeq**			**CNVnator**		
	**CNV segment counts**	**1 kb**	**5 kb**	**10kb**	**1 kb**	**5 kb**	**10 kb**	**1 kb**	**5 kb**	**10 kb**
Gain	Correct detection	10	10	10	7	10	10	6	9	10
Loss or absent	Correct detection	9	10	10	7	4	6	8	10	10
Normal	False positives	0	0	3	1	7	6	436	466	399

**Table 5:** Overlap rate of true CNV segments and detected CNV segments in simulation study 3, comparing between m-HMM with and without change point adjustment

**Overlap rate**	**CNV segment length**		
	**1 kb**	**5 kb**	**10 kb**
m-HMM without adj.	0.724	0.912	0.902
m-HMM	0.859	0.971	0.927

The overlap rate is evaluated using the overlap length divided by the true CNV segment length.

Table 5 lists the overlap rates of true CNV segments and detected CNV segments by m-HMM with and without change point adjustment. The overlap rate is calculated using the overlap genomic length divided by the true CNV genomic length. We see that among all the corrected detected CNV segments, the overlap rates are quite high (over 70%). Also, the change point adjustment makes the overlap rate even higher (over 85%).

### Application

We applied the proposed m-HMM to compare the sequence data from two maize genotypes: B73 and Mo17. The previously sequenced B73 genome [3] was used as the reference genome with the Mo17 genome as the target. The original data was provided by Gore et al. [26], and was pre-processed by the lab of Schnable. In the data preparation stage, 44 base sequence reads from each of the B73 and Mo17 genomes were aligned to the reference B73 genome. We obtained 4.3 million aligned reads from B73 and 1.54 million aligned reads from Mo17, and 2.3 million genomic positions on the reference B73 genome had positive read counts. Using the m-HMM, we found 1096 segments of 2000 bases or longer that have copy number variations that are at least 2 fold increasing/decreasing, among which, Mo17 has 14 segments with copy number gain state, 835 segments with copy number loss state and 247 segments with absent state, compared to B73.

**Figure 2:**
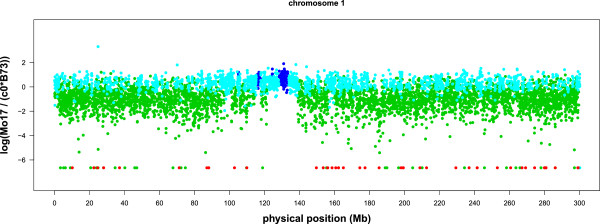
**Log ratio between the counts of B73 and Mo17 on chromosome 1.** Each point represents a window.

**Figure 3:**
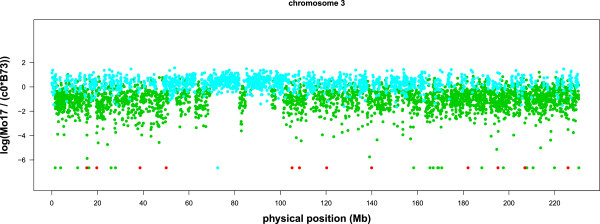
**Log ratio between the counts of B73 and Mo17 on chromosome 3.** Each point represents a window.

**Figure 4:**
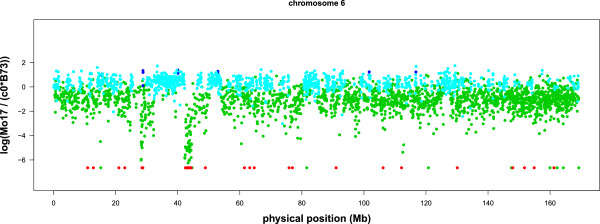
**Log ratio between the counts of B73 and Mo17 on chromosome 6.** Each point represents a window.

**Figure 5:**
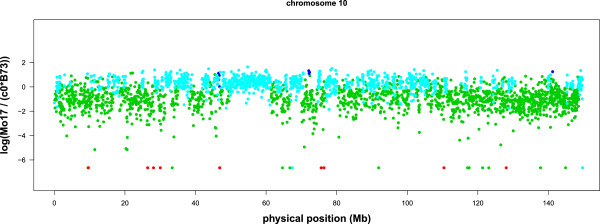
**Log ratio between the counts of B73 and Mo17 on chromosome 10.** Each point represents a window.

We present the window based m-HMM result for chromosome 1, 3, 6 and 10 in Figures 2, 3, 4 and 5. In each figure, the horizontal axis represents the genomic location on the reference B73 genome (in million bases, or Mb), and the vertical axis represents the log of the normalized count ratio between Mo17 and B73, i.e., $\log\left(\frac{\text{Mo17}}{c_{0}\text{B73}}\right)$ with the log ratio set to -7 when the Mo17 count is zero. The blue, teal, green and red colors represent copy number gain, normal, copy number loss and absent state, respectively.

There are several large segments with few copy number variations between Mo17 and B73. They are 121.3 Mb ∼ 130 Mb on chromosome 1 (Figure 2), 69.2 Mb ∼ 82.0 Mb and 84.8 Mb ∼ 95.9 Mb on chromosome 3 (Figure 3), and 49.5 Mb ∼ 61.5 Mb on chromosome 10 (Figure 5). These results are concordant with previous results using aCGH data [6,7].

The most extreme copy number loss or absent state detected on Mo17 is in chromosome 6 (Figure 4), located from 42.2 million to 46.2 million bases on the reference genome. Within this segment, the reference genome B73 has 8152 total read counts, and the sample genome Mo17 has 542 total read counts. The copy number ratio is about 0.066, or 0.13 after taking into account the normalization factor *c*_0_. Table 6 demonstrates the m-HMM segmentation result between 35.3 million and 57.0 million bases. Two other long segments with copy number loss or absent state in chromosome 6 are from 26.5 million to 28.8 million bases, and from 47.8 million to 49.7 million bases. These identifications are also concordant with the result from other studies that compared DNA sequences between B73 and Mo17 using aCGH data [6,7].

**Table 6:** The m-HMM segmentation result for chromosome 6 from 35.3 Mb to 57.0 Mb

**Start Position**	**End Position**	**Mo17 Read counts**	**B73 Read counts**	**Mo17/B73**^**∗**^
35,274,511	36,847,009	1,511	2291	1.33
36,847,028	36,986,070	81	242	0.67
36,988,155	39,227,681	2,375	3850	1.21
39,227,711	39,392,612	53	294	0.36
39,392,816	39,998,180	605	810	1.51
39,998,210	40,070,876	114	95	2.43
40,071,001	42,219,831	1,978	2751	1.45
42,221,884	42,272,394	3	305	0.01
42,273,061	42,279,935	0	37	0.00
42,281,369	42,467,432	6	484	0.02
42,467,722	42,510,527	0	199	0.00
42,520,362	42,588,228	10	134	0.15
42,589,445	42,592,668	0	16	0.00
42,600,850	42,949,605	41	734	0.11
42,957,788	43,020,977	0	213	0.00
43,021,808	43,021,808	1	0	50.00
43,021,875	43,034,674	0	20	0.00
43,050,012	43,156,464	7	214	0.06
43,159,602	43,239,996	0	110	0.00
43,240,069	43,240,069	4	1	8.11
43,240,496	43,253,531	0	25	0.00
43,270,468	43,367,135	27	396	0.13
43,382,243	43,436,720	0	211	0.00
43,437,634	43,437,634	1	0	50.00
43,437,664	43,438,342	0	19	0.00
43,440,089	43,466,314	8	146	0.11
43,466,499	43,502,281	0	183	0.00
43,502,441	43,502,441	1	0	50.00
43,504,419	43,516,326	0	3	0.00
43,532,757	43,802,344	29	815	0.077
43,802,387	43,809,486	0	214	0.00
43,809,952	43,809,952	4	0	50.00
43,809,982	43,811,684	0	47	0.00
43,812,791	43,828,685	1	128	0.01
43,833,620	43,862,804	0	121	0.00
43,863,025	43,863,025	1	0	50.00
43,866,525	43,959,900	0	164	0.00
43,959,976	43,960,274	2	10	0.40
43,960,449	43,984,663	0	95	0.00
43,991,670	44,242,537	24	498	0.09
44,247,904	44,297,744	0	50	0.00
44,297,961	44,297,961	1	0	50.00
44,299,769	44,341,066	0	252	0.00
44,341,857	46,215,928	371	2308	0.32
46,215,958	46,834,832	572	895	1.29
46,835,472	47,130,192	74	545	0.27
47,130,202	47,668,306	642	833	1.56
47,681,491	47,739,876	18	72	0.50
47,740,273	47,740,273	0	2	0.00
47,746,723	48,880,371	283	1356	0.42
48,880,568	48,923,056	0	51	0.00
48,923,160	49,722,664	213	1128	0.38
49,722,844	50,132,724	231	298	1.57
50,132,929	50,239,093	28	119	0.47
50,260,756	52,852,085	2,487	3567	1.41
52,852,089	52,958,397	155	130	2.41
52,958,403	53,950,690	918	1453	1.28
53,951,899	56,137,418	895	3196	0.56
56,142,451	56,472,218	296	570	1.05
56,472,614	56,863,324	102	570	0.36
56,866,117	57,022,366	123	212	1.17

The first two columns are the start position and the end position of each of the CNV segments. Column 3 and column 4 are the read counts for Mo17 and B73 between the start position and the end position, respectively. The last column is the normalized read count ratio between Mo17 and B73, i.e., $\text{log}\left(\frac{\text{Mo17}}{{\text{c}}_{\text{0}}\cdot \text{B73}}\right)$ for that CNV segment.

*The last column Mo17/B73 represents the normalized ratio of read counts between Mo17 and B73 for the genomic region between the start position and the end position. The normalization factor *c*_0_ = 0.493.

Table 7 lists the detected CNV segments that are longer than 2 Mb and show greater than 2-fold normalized changes between B73 and Mo17. Plots for all the maize chromosomes are provided in the Additional file 2: Appendix 2.

**Table 7:** This table lists the detected CNV segments that are longer than 2 Mb and presents greater than 2-fold normalized read count differences between B73 and Mo17

	**Start Position**	**End Position**	**Mo17/B73**^**∗**^
chr 1	7,674,194	10,130,535	0.47
	73,051,891	76,150,311	0.49
	78,765,566	80,973,937	0.40
	106,020,503	108,085,310	0.46
	183,346,370	185,514,363	0.34
	185,561,332	187,744,738	0.34
	206,016,332	208,795,988	0.49
chr 2	14,327,370	17,154,616	0.49
	43,421,667	45,665,032	0.47
	53,360,846	57,062,635	0.48
	101,464,135	103,592,856	0.37
	173,361,552	175,485,424	0.46
	183,250,632	186,406,359	0.46
	209,163,585	211,227,635	0.48
	211,597,012	214,704,617	0.48
chr 3	3,653,572	5,790,383	0.44
	45,465,224	48,749,064	0.40
	82,020,005	84,053,318	0.48
	176,248,175	179,361,747	0.48
chr 4	645	2,454,064	0.45
	23,025,833	25,854,581	0.48
	136,792,400	140,387,061	0.36
	140,502,832	143,245,529	0.49
	146,672,844	148,733,733	0.44
	239,597,061	242,255,833	0.48
	243,186,165	245,433,834	0.49
chr 5	753,941	4,311,600	0.48
	5,201,605	7,723,585	0.48
	11,328,383	14,574,771	0.49
	16,611,265	19,032,650	0.44
	68,583,278	71,949,086	0.40
	156,325,001	158,697,338	0.47
	168,069,569	170,816,075	0.39
	176,121,726	179,598,151	0.43
	191,459,521	195,013,031	0.48
chr 6	25,898,308	28,476,332	0.33
	70,440,134	72,858,938	0.46
	76,981,074	80,570,101	0.46
	85,620,416	87,659,132	0.48
	102,350,617	104,422,628	0.49
	154,917,250	159,407,299	0.47
	162,488,577	165,722,159	0.48
chr 7	18,304,500	22,673,932	0.49
	25,498,467	30,547,169	0.47
	78,344,496	81,198,663	0.40
	110,502,115	113,262,773	0.49
	117,913,056	119,946,052	0.49
	120,044,241	122,233,553	0.49
chr 8	20,108,771	23,447,168	0.43
	160,011,805	166,514,647	0.45
	167,430,533	169,880,960	0.45
chr 9	38,523,842	40,732,642	0.41
	46,734,889	49,382,812	0.38
	61,499,201	64,577,206	0.39
	83,491,462	88,345,837	0.46
	118,555,633	121,557,270	0.49
	143,196,239	145,630,853	0.45
	145,813,631	150,079,829	0.46
chr 10	20,262,176	24,734,243	0.48
	39,046,734	41,852,436	0.46
	72,370,857	74,553,412	0.42
	111,247,775	116,238,001	0.46
	117,013,634	120,679,932	0.49
	121,011,209	124,203,304	0.49
	130,590,451	139,841,271	0.46

The last column Mo17/B73 represents the normalized ratio of read counts between Mo17 and B73 for the genomic region between the start position and the end position. The normalization factor *c*_0_ = 0.493.

Several challenging aspects of this application should be noted when interpreting our results. First, there exists a high level of divergence between the target genome Mo17 and the reference genome B73. Because of the high divergence, it is difficult to map the target reads to the reference genome in some regions. This might partially explain the reason that many of the identified CNVs are classified as copy number loss or absent in Mo17 relative to B73. Moreover, the short read length of 44 bp in this data set makes it even more difficult to correctly map the target reads to the reference genome. Secondly, the average coverage depth is also very low (less than 1*X*). This makes it difficult to identify small CNV segments. Although higher sequencing depth provides better CNV detection accuracy, Sims et al. [27] pointed out that reasonable detections can still be obtained for large CNVs when the coverage is at least 0.1*X*.

With fast developing sequencing technologies, higher coverage depths and longer reads are available in may of today’s sequencing data sets. Reads with 100 bp are standard, and it is common to see 150 to 300 bp in Illumina reads. Also, coverage depths of 30*X* and higher are becoming mainstream. With longer reads, more precise mapping and higher coverage depth, the m-HMM is expected to provide better CNV detections with higher accuracy.

It is also important to note that DNA sequencing technologies are potentially affected by biases that arise from several biophysical and chemical features [28]. For example, favorable levels of GC-content could amplify the coverage of the genome so as to affect CNV detections. Many recent methods have addressed GC-content bias in CNV detections. Risso et al. [29] and Janevski, et al. [28] reviewed GC-content normalization tools and methods that are currently being used. Our method is based on direct comparison of two genomes within genomic windows. As long as the within-window GC content remains similar across genomes, variation in GC content across the genomes should not bias our m-HMM results.

## Conclusions

In this paper, we proposed a new methodology to detect CNVs in the DNA sequences from two genotypes using next generation sequencing data. We used a hidden Markov model incorporating a mixture emission probability model to identify the copy number variation change points. The simulations study suggests that the m-HMM has better sensitivities and specificities in CNV identifications comparing to other methodologies.

The proposed m-HMM was applied to compare the two maize inbred lines B73 and Mo17, and identified CNV change points of the target sequence Mo17 relative to the reference sequence B73. The result of the m-HMM is concordant with previous genomic studies using aCGH data by Springer et al. [7] and Belo et al. [6].

In addition, the m-HMM can be used to compare two genotypes when only one sample of target reads and only one sample of reference reads are available. Many other existing methods require multiple read samples for both target and reference genotypes. Thus, the m-HMM approach we have proposed may be especially useful when financial constraints limit data collection.

## Availability of the software and supporting data set

The m-HMM software and an example data set are available in the webpage https://www.stt.msu.edu/users/hengwang/mHMM.html. The maize sequencing data set supporting the results of this article can be downloaded through the link ftp://ftp.ensemblgenomes.org/pub/plants/release-10/fasta/zea_mays/dna/.

## Competing interests

The authors declare that they have no competing interests.

## Authors’ contributions

HW designed the model and the algorithm, carried out the simulation studies and data application, and drafted the manuscript. DN supervised the study, participated in the model design, and helped draft the manuscript. KY provided the data pre-processing and the model testing. KY also simulated the data set for the third simulation study. All authors read and approved the final manuscript.

## Supplementary Material

Additional file 1 **Appendix 1 – More Details in the EM Algorithm **[30]**.** Click here for file

Additional file 2 Appendix 2 – Maize CNV Detection Results. Click here for file
