# Supplementary material for: Copy number variation detection using next generation sequencing read counts
Source: BMC Bioinformatics. 2014 Apr 14;15:109. doi: 10.1186/1471-2105-15-109 (PMC4021345; doi:10.1186/1471-2105-15-109)
Supplement: Additional file 1 — Appendix 1 – More Details in the EM Algorithm [30]. [file 1471-2105-15-109-S1.pdf]

Additional file 1: Appendix 1

More Details in the EM Algorithm

The EM algorithm is an iterative procedure to find the parameter value $\boldsymbol{\theta}$ that maximizes the likelihood function $\mathscr{L}(\boldsymbol{\theta}|\boldsymbol{u})$. The observed data likelihood function is

$$\mathscr{L}_{(\text{obs})} = \mathscr{L}(\boldsymbol{\theta}|\boldsymbol{u}) = \sum_{\boldsymbol{s}} \pi_{s_1} \prod_{w=1}^{W-1} a_{s_w s_{w+1}}(w) \prod_{w=1}^{W} P(\boldsymbol{u}_w|\boldsymbol{s}, \alpha, \beta, c_0, \boldsymbol{q}, \boldsymbol{v}).$$

The complete data likelihood function is

$$\mathscr{L}_{(\text{comp})} = \mathscr{L}(\boldsymbol{\theta}|\boldsymbol{u}, \boldsymbol{s}) = \pi_{s_1} \prod_{w=1}^{W-1} a_{s_w s_{w+1}}(w) \prod_{w=1}^{W} P(\boldsymbol{u}_w|\boldsymbol{s}, \alpha, \beta, c_0, \boldsymbol{q}, \boldsymbol{v}).$$

1. The E step:

   The E step is to evaluate the expectation of the complete data log-likelihood with respect to the conditional distribution of the hidden states $\boldsymbol{S}$, given the observation $\boldsymbol{u}$, and assuming the parameter vector $\boldsymbol{\theta}$ is equal to $\boldsymbol{\theta}^{(m)}$, the value of $\boldsymbol{\theta}$ determined in iteration m of the algorithm: $E_{\boldsymbol{S}|\boldsymbol{u},\boldsymbol{\theta}^{(m)}}(\ell(\boldsymbol{\theta}|\boldsymbol{u}, \boldsymbol{s}))$, where ℓ is an abbreviation for $\log \mathscr{L}$.

   The expectation with respect to the conditional probability of the hidden states, given the observations $\boldsymbol{u}$ and the parameters $\boldsymbol{\theta}^{(m)}$ obtained from the mth iter-

ation is

$$
\begin{aligned}
&E_{\boldsymbol{S}|\boldsymbol{u},\boldsymbol{\theta}^{(m)}}(\ell(\boldsymbol{\theta}|\boldsymbol{u},\boldsymbol{s}))\\
&= \sum_{\boldsymbol{s}} P(\boldsymbol{S}=\boldsymbol{s}|\boldsymbol{u},\boldsymbol{\theta}^{(m)})\Big[\log \pi_{s_1} + \sum_{w=1}^{W-1}\log\big(a_{s_w s_{w+1}}(w)\big) + \sum_{w=1}^{W}\log P(\boldsymbol{u}_w|\boldsymbol{s},\alpha,\beta,c_0,\boldsymbol{q},\boldsymbol{v})\Big]\\
&= \sum_{\boldsymbol{s}} P(\boldsymbol{S}=\boldsymbol{s}|\boldsymbol{u},\boldsymbol{\theta}^{(m)})\log \pi_{s_1} \qquad (1)\\
&\quad + \sum_{\boldsymbol{s}} P(\boldsymbol{S}=\boldsymbol{s}|\boldsymbol{u},\boldsymbol{\theta}^{(m)})\sum_{w=1}^{W-1}\log\big(a_{s_w s_{w+1}}(w)\big) \qquad (2)\\
&\quad + \sum_{\boldsymbol{s}} P(\boldsymbol{S}=\boldsymbol{s}|\boldsymbol{u},\boldsymbol{\theta}^{(m)})\sum_{w=1}^{W}\log P(\boldsymbol{u}_w|\boldsymbol{s},\alpha,\beta,c_0,\boldsymbol{q},\boldsymbol{v}) \qquad (3)\\
&= \sum_{k=1}^{4} P_k(1)\log(\pi_k) + \sum_{w=1}^{W-1}\sum_{k=1}^{4}\sum_{l=1}^{4} P_{kl}(w)\log(a_{kl}(w))\\
&\quad + \sum_{w=1}^{W}\sum_{k=1}^{4} P_k(w)\log P(\boldsymbol{u}_w|\boldsymbol{S}_w=k,\alpha,\beta,c_0,\boldsymbol{q},\boldsymbol{v}),
\end{aligned}
$$

where $P_k(w) = P(S_w = k|\boldsymbol{u},\boldsymbol{\theta}^{(m)})$, $P_{kl}(w) = P(S_w = k, S_{w+1} = l|\boldsymbol{u},\boldsymbol{\theta}^{(m)})$ at the mth iteration, and the equality following (3) can be demonstrated as follows. First, note that expression (1) reduces to

$$
\begin{aligned}
&\sum_{\boldsymbol{s}}\sum_{k=1}^{4} P(\boldsymbol{S}=\boldsymbol{s}|\boldsymbol{u},\boldsymbol{\theta})\mathbb{I}_{\{S_1=k\}}\log(\pi_k)\\
&= \sum_{k=1}^{4}\sum_{\boldsymbol{s}} P(\boldsymbol{S}=\boldsymbol{s}|\boldsymbol{u},\boldsymbol{\theta})\mathbb{I}_{\{S_1=k\}}\log(\pi_k)\\
&= \sum_{k=1}^{4} P_k(1)\log(\pi_k),
\end{aligned}
$$

We omit the superscript (m) here for simplicity. Also we omit (m) through the end of the E-step description. But the probabilities that appear here do depend on the current $\boldsymbol{\theta}$ value and change with iterations.

Next, (2) may be simplified as

$$\begin{aligned}
&\sum_{\boldsymbol{s}} P(\boldsymbol{S}=\boldsymbol{s}|\boldsymbol{u},\boldsymbol{\theta}) \sum_{w=1}^{W-1} \log\left(a_{s_w s_{w+1}}(w)\right) \\
&= \sum_{\boldsymbol{s}} P(\boldsymbol{S}=\boldsymbol{s}|\boldsymbol{u},\boldsymbol{\theta}) \sum_{w=1}^{W-1}\sum_{k=1}^{4}\sum_{l=1}^{4} \mathbb{I}_{\{S_w=k\}}\mathbb{I}_{\{S_{w+1}=l\}} \log(a_{kl}(w)) \\
&= \sum_{w=1}^{W-1}\sum_{k=1}^{4}\sum_{l=1}^{4} \left[\sum_{\boldsymbol{s}} P(\boldsymbol{S}=\boldsymbol{s}|\boldsymbol{u},\boldsymbol{\theta})\mathbb{I}_{\{S_w=k\}}\mathbb{I}_{\{S_{w+1}=l\}}\right] \log(a_{kl}(w)) \\
&= \sum_{w=1}^{W-1}\sum_{k=1}^{4}\sum_{l=1}^{4} P_{kl}(w)\log(a_{kl}(w)).
\end{aligned}$$

Finally, expression (3) may be written as

$$\begin{aligned}
&\sum_{\boldsymbol{s}} P(\boldsymbol{S}=\boldsymbol{s}|\boldsymbol{u},\boldsymbol{\theta}) \sum_{w=1}^{W} \log P(\boldsymbol{u}_w|\boldsymbol{s},\alpha,\beta,c_0,\boldsymbol{q},\boldsymbol{v}) \\
&= \sum_{w=1}^{W}\sum_{k=1}^{4}\sum_{\boldsymbol{s}} P(\boldsymbol{S}=\boldsymbol{s}|\boldsymbol{u},\boldsymbol{\theta})\mathbb{I}_{\{S_w=k\}} \log P(\boldsymbol{u}_w|S_w=k,\alpha,\beta,c_0,\boldsymbol{q},\boldsymbol{v}) \\
&= \sum_{w=1}^{W}\sum_{k=1}^{4} P_k(w)\log P(\boldsymbol{u}_w|S_w=k,\alpha,\beta,c_0,\boldsymbol{q},\boldsymbol{v}).
\end{aligned}$$

The numeric values of $P_k(w) = \frac{P(S_w=k,\boldsymbol{u}|\boldsymbol{\theta})}{P(\boldsymbol{u}|\boldsymbol{\theta})}$ and $P_{kl}(w) = \frac{P(S_w=k,S_{w+1}=l,\boldsymbol{u}|\boldsymbol{\theta})}{P(\boldsymbol{u}|\boldsymbol{\theta})}$ can be evaluated using the forward-backward algorithm, which was introduced by Rabiner and Juang (1986). The forward probability $f_k(w)$ is defined as the probability of having state k at window w, and having the observations $\{\boldsymbol{u}_1,\ldots,\boldsymbol{u}_w\}$ from window 1 to window w, given the parameter $\boldsymbol{\theta}$, i.e., $f_k(w) = P(\boldsymbol{u}_1,\ldots,\boldsymbol{u}_w,S_w=k|\boldsymbol{\theta})$. The backward probability $b_k(w)$ is defined as the probability of having the observations $\{\boldsymbol{u}_{w+1},\ldots,\boldsymbol{u}_W\}$ from window $w+1$ to window W, given the state k at window w, the observations from window 1 to window w, and the parameter $\boldsymbol{\theta}$, i.e., $b_k(w) = P(\boldsymbol{u}_{w+1},\ldots,\boldsymbol{u}_W|S_w = k,\boldsymbol{u}_1,\ldots,\boldsymbol{u}_w,\boldsymbol{\theta})$. The forward and backward probabilities can be obtained using recursions: $f_k(1) = \pi_k P(\boldsymbol{u}_1|S_1=k,\boldsymbol{\theta})$, $b_k(W)=1$, $f_k(w) = \sum_{l=1}^4 f_l(w-1)a_{lk}(w-1)P(\boldsymbol{u}_w|S_w=k,\boldsymbol{\theta})$ for $w=2,\ldots,W$, and $b_k(w) = \sum_{l=1}^4 a_{kl}(w)P(\boldsymbol{u}_{w+1}|S_{w+1}=l,\boldsymbol{\theta})b_l(w+1)$ for $w=W-1,\ldots,1$.

Consequently,

$$\begin{aligned}
&P(S_w = k, \boldsymbol{u}|\boldsymbol{\theta}) \\
&= P(\boldsymbol{u}_1, \ldots, \boldsymbol{u}_W, S_w = k|\boldsymbol{\theta}) \\
&= P(\boldsymbol{u}_1, \ldots, \boldsymbol{u}_w, S_w = k|\boldsymbol{\theta}) P(\boldsymbol{u}_{w+1}, \ldots, \boldsymbol{u}_W | S_w = k, \boldsymbol{u}_1, \ldots, \boldsymbol{u}_w, \boldsymbol{\theta}) \\
&= f_k(w) b_k(w),
\end{aligned}$$

and

$$\begin{aligned}
&P(S_w = k, S_{w+1} = l, \boldsymbol{u}|\boldsymbol{\theta}) \\
&= P(\boldsymbol{u}_1, \ldots, \boldsymbol{u}_w, S_w = k|\boldsymbol{\theta}) \cdot P(\boldsymbol{u}_{w+1}, S_{w+1} = l | S_w = k, \boldsymbol{u}_1, \ldots, \boldsymbol{u}_w, \boldsymbol{\theta}) \\
&\quad \cdot P(\boldsymbol{u}_{w+2}, \ldots, \boldsymbol{u}_W | S_w = k, S_{w+1} = l, \boldsymbol{u}_1, \ldots, \boldsymbol{u}_{w+1}, \boldsymbol{\theta}) \\
&= f_k(w) P(\boldsymbol{u}_{w+1}, S_{w+1} = l | S_w = k, \boldsymbol{\theta}) b_l(w+1) \\
&= f_k(w) a_{kl}(w) P(\boldsymbol{u}_{w+1} | S_{w+1} = l, \boldsymbol{\theta}) b_l(w+1).
\end{aligned}$$

2. The M step:

   The M step of EM algorithm is to find the value of $\boldsymbol{\theta}$ that makes $E_{\boldsymbol{S}|\boldsymbol{u},\boldsymbol{\theta}^{(m)}}(\ell(\boldsymbol{\theta}|\boldsymbol{u}, \boldsymbol{s}))$ obtain the maximum. This maximizing value is the updated parameter $\boldsymbol{\theta}^{(m+1)}$ for the $(m+1)^{\text{th}}$ iteration.

$$
\begin{aligned}
&E_{\boldsymbol{S}|\boldsymbol{u},\boldsymbol{\theta}^{(m)}}(\ell(\boldsymbol{\theta}|\boldsymbol{u},\boldsymbol{s}))\\
&= \sum_{\boldsymbol{s}} P(\boldsymbol{S}|\boldsymbol{u},\boldsymbol{\theta})\Big[\log \pi_{s_1} + \sum_{w=1}^{W-1}\log\big(a_{s_w s_{w+1}}(w)\big) + \sum_{w=1}^{W}\log P(\boldsymbol{u}_w|\boldsymbol{s},\alpha,\beta,c_0,\boldsymbol{q},\boldsymbol{v})\Big]\\
&= \sum_{k=1}^{4} P_k(1)\log(\pi_k) + \sum_{w=1}^{W-1}\sum_{k=1}^{4}\sum_{l=1}^{4} P_{kl}(w)\log(a_{kl}(w))\\
&\quad + \sum_{w=1}^{W}\sum_{k=1}^{4} P_k(t)\log P(\boldsymbol{u}_w|S_w=k,\alpha,\beta,c_0,\boldsymbol{q},\boldsymbol{v})\\
&= \sum_{k=1}^{4} P_k(1)\log(\pi_k) + \sum_{w=1}^{W-1}\sum_{k=1}^{4} P_{kk}(w)\log\left(1-\Big(\sum_{l'\neq k} p_{kl'}\Big)\Big(1-e^{-\rho d_w}\Big)\right)\\
&\quad + \sum_{w=1}^{W-1}\sum_{k=1}^{4}\sum_{l\neq k} P_{kl}(w)\log\left(p_{kl}\Big(1-e^{-\rho d_w}\Big)\right)\\
&\quad + \sum_{w=1}^{W}\sum_{k=1}^{4} P_k(w)\log P(\boldsymbol{u}_w|S_w=k,\alpha,\beta,c_0,\boldsymbol{q},\boldsymbol{v})\\
&\triangleq G_1(\pi_k) + G_2(\boldsymbol{p},\rho) + G_3(\boldsymbol{p},\rho) + G_4(\boldsymbol{s},\alpha,\beta,c_0,\boldsymbol{q},\boldsymbol{v}).
\end{aligned}
$$

Equating to zero the derivative of $E_{\boldsymbol{S}|\boldsymbol{u},\boldsymbol{\theta}^{(m)}}(\ell(\boldsymbol{\theta}|\boldsymbol{u},\boldsymbol{s}))$ with respect to p_{kl} yields

$$
\begin{aligned}
&\frac{\partial G_2(\boldsymbol{p},\rho)}{\partial p_{kl}} + \frac{\partial G_3(\boldsymbol{p},\rho)}{\partial p_{kl}} \triangleq 0 \quad (k,l=1,\ldots,4; l\neq k)\\
\Rightarrow\quad &\sum_{w=1}^{W-1}\frac{(1-e^{-\rho d_w})P_{kk}(w)}{1-(1-e^{-\rho d_w})\sum_{l\neq k}p_{kl}} = \sum_{w=1}^{W-1}\frac{P_{kl}(w)}{p_{kl}} \quad (k,l=1,\ldots,4; l\neq k)\\
\Rightarrow\quad &\sum_{w=1}^{W-1}\frac{P_{k1}(w)}{p_{k1}} = \ldots = \sum_{w=1}^{W-1}\frac{P_{k4}(w)}{p_{k4}} = \sum_{w=1}^{W-1}\frac{(1-e^{-\rho d_w})P_{kk}(w)}{1-(1-e^{-\rho d_w})\sum_{l\neq k}p_{kl}}
\end{aligned}
$$

for $k,l=1,\ldots,4$ and $l\neq k$.

Letting $\sum_{w=1}^{W-1}\frac{P_{kl}(w)}{p_{kl}} = h_k$, $k=1,\ldots,4$, we find the value of h_k that maximizes

$$
\begin{aligned}
&\sum_{w=1}^{W-1} P_{kk}(w)\log\left(1-\Big(\frac{\sum_{l'\neq k}\sum_{w=1}^{W-1}P_{kl'}(w)}{h_k}\Big)\Big(1-e^{-\rho d_w}\Big)\right)\\
&\quad + \sum_{w=1}^{W-1}\sum_{l\neq k} P_{kl}(w)\log\left(\frac{\sum_{w=1}^{W-1}P_{kl}(w)}{h_k}\Big(1-e^{-\rho d_w}\Big)\right)
\end{aligned}
$$

for each k with ρ initially fixed at its value from the previous EM iteration ($\rho^{(m)}$). Then a new $\boldsymbol{p}$ value can be obtained by $p_{kl}(w) = \frac{\sum_{w=1}^{W-1}P_{kl}(w)}{h_k}$, $k,l=1\ldots,4$,

$l \neq k$. Now, an updated value of ρ can be obtained by directly maximizing $G_2(\boldsymbol{p}, \rho) + G_3(\boldsymbol{p}, \rho)$ with respect to ρ, using the new $\boldsymbol{p}$ value.

After obtaining a pair of values of $\boldsymbol{p}$ and ρ that maximize $G_2(\boldsymbol{p}, \rho) + G_3(\boldsymbol{p}, \rho)$, we estimate the values for $\alpha, \beta, \boldsymbol{q}$ and $\boldsymbol{v}$ by maximizing G_4. EM iteration continues until all parameter values getting converge, at which point we obtain an updated $\boldsymbol{\theta}^{(m+1)}$ value.

Derivation for Equation (4)

Here we provide a detailed derivation for equation (4). Conditional on the hidden copy number state for window w, the joint distribution for the target and the reference read counts at window w is

$$
\begin{aligned}
& P^{(*)}(U_w^{[t]} = u_w^{[t]}, U_w^{[r]} = u_w^{[r]} | S_w = k, \boldsymbol{\theta}) \\
= & \int_0^\infty P(U_w^{[t]} = u_w^{[t]}, U_w^{[r]} = u_w^{[r]}, \lambda_w^{[r]} | S_w = k, \boldsymbol{\theta}) d\lambda_w^{[r]} \\
= & \int_0^\infty P(U_w^{[t]} = u_w^{[t]}, U_w^{[r]} = u_w^{[r]} | \lambda_w^{[r]}, S_w = k, \boldsymbol{\theta}) P(\lambda_w^{[r]} | S_w = k) d\lambda_w^{[r]} \\
= & \int_0^\infty P(U_w^{[t]} = u_w^{[t]} | \lambda_w^{[r]}, S_w = k, \boldsymbol{\theta}) P(U_w^{[r]} = u_w^{[r]} | \lambda_w^{[r]}, S_w = k, \boldsymbol{\theta}) P(\lambda_w^{[r]} | S_w = k) d\lambda_w^{[r]}
\end{aligned}
$$

According to (3),

$$
U_w^{[t]} | (\lambda_w^{[r]}, S_w = k) \sim \sum_{j=1}^{4} q_{kj} \text{Poisson}(v_{kj} c_0 \lambda_w^{[r]}),
$$

we have

$$
\begin{aligned}
& P(U_w^{[t]} = u_w^{[t]} | \lambda_w^{[r]}, S_w = k, \boldsymbol{\theta}) \\
= & \sum_{j=1}^{4} q_{kj} \frac{(v_{kj} c_0 \lambda_w^{[r]})^{u_w^{[t]}} e^{-v_{kj} c_0 \lambda_w^{[r]}}}{u_w^{[t]}!}
\end{aligned}
$$

So

$$
\begin{aligned}
&P^{(*)}(U_w^{[t]} = u_w^{[t]}, U_w^{[r]} = u_w^{[r]} | S_w = k, \boldsymbol{\theta}) \\
&= \sum_{j=1}^{4} q_{kj} \int_0^\infty \frac{(v_{kj} c_0 \lambda_w^{[r]})^{u_w^{[t]}} e^{-v_{kj} c_0 \lambda_w^{[r]}}}{u_w^{[t]}!} \cdot \frac{(\lambda_w^{[r]})^{u_w^{[r]}} e^{-\lambda_w^{[r]}}}{u_w^{[r]}!} \cdot \frac{\beta^\alpha (\lambda_w^{[r]})^{\alpha-1} e^{-\beta \lambda_w^{[r]}}}{\Gamma(\alpha)} d\lambda_w^{[r]} \\
&= \sum_j q_{kj} \frac{\Gamma(u_w^{[t]} + u_w^{[r]} + \alpha)(v_{kj} c_0)^{u_w^{[t]}} \beta^\alpha}{\Gamma(\alpha) u_w^{[r]}! u_w^{[t]}! (v_{kj} c_0 + 1 + \beta)^{u_w^{[t]} + u_w^{[r]} + \alpha}} \\
&\quad \int_0^\infty \frac{(v_{kj} c_0 + 1 + \beta)^{u_w^{[t]} + u_w^{[r]} + \alpha} (\lambda_w^{[r]})^{u_w^{[r]} + u_w^{[t]} + \alpha - 1} e^{-(v_{kj} c_0 + \beta + 1)} \lambda_w^{[r]}}{\Gamma(u_w^{[t]} + u_w^{[r]} + \alpha)} d\lambda_w^{[r]}
\end{aligned}
$$

The last integral is a integral of a Gamma distribution with parameters $u_w^{[r]} + u_w^{[t]} + \alpha$ and $v_{kj} c_0 + 1 + \beta$ so is equal to 1. Then we have

$$
\begin{aligned}
&P^{(*)}(U_w^{[t]} = u_w^{[t]}, U_w^{[r]} = u_w^{[r]} | S_w = k, \boldsymbol{\theta}) \\
&= \sum_j q_{kj} \frac{\Gamma(u_w^{[t]} + u_w^{[r]} + \alpha)(v_{kj} c_0)^{u_w^{[t]}} \beta^\alpha}{\Gamma(\alpha) u_w^{[r]}! u_w^{[t]}! (v_{kj} c_0 + 1 + \beta)^{u_w^{[t]} + u_w^{[r]} + \alpha}},
\end{aligned}
$$

which is equation (4).
