# Supplementary figures and images for: Copy number variation detection using next generation sequencing read counts
Source: BMC Bioinformatics. 2014 Apr 14;15:109. doi: 10.1186/1471-2105-15-109 (PMC4021345; doi:10.1186/1471-2105-15-109)

## Additional file 2: Appendix 2

### Maize CNV Detection Results

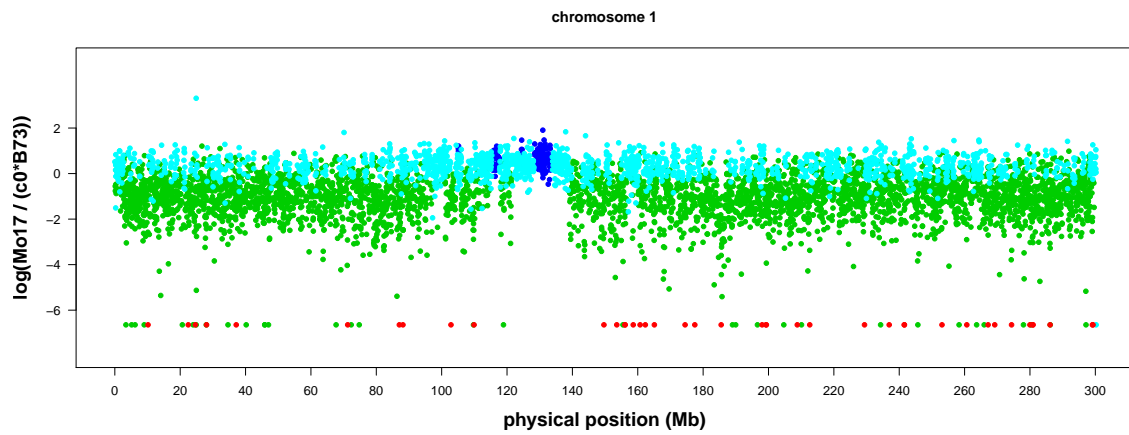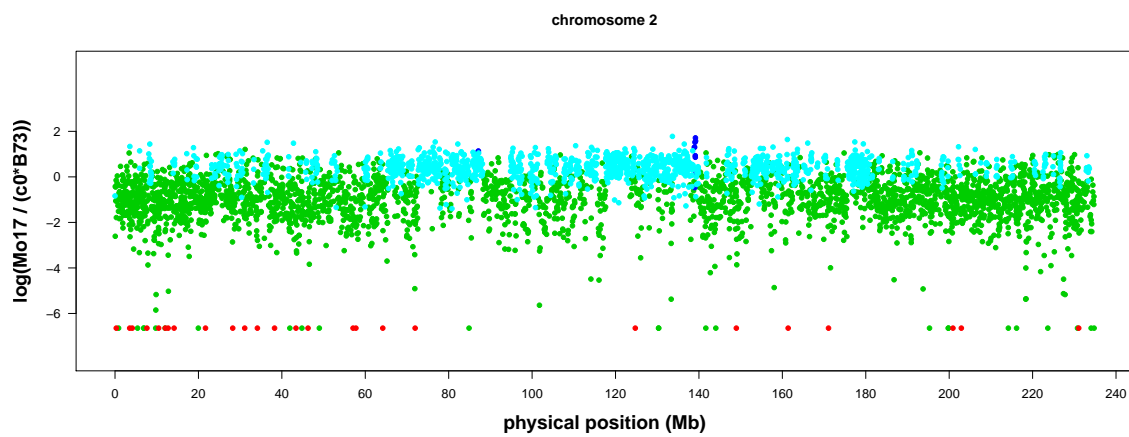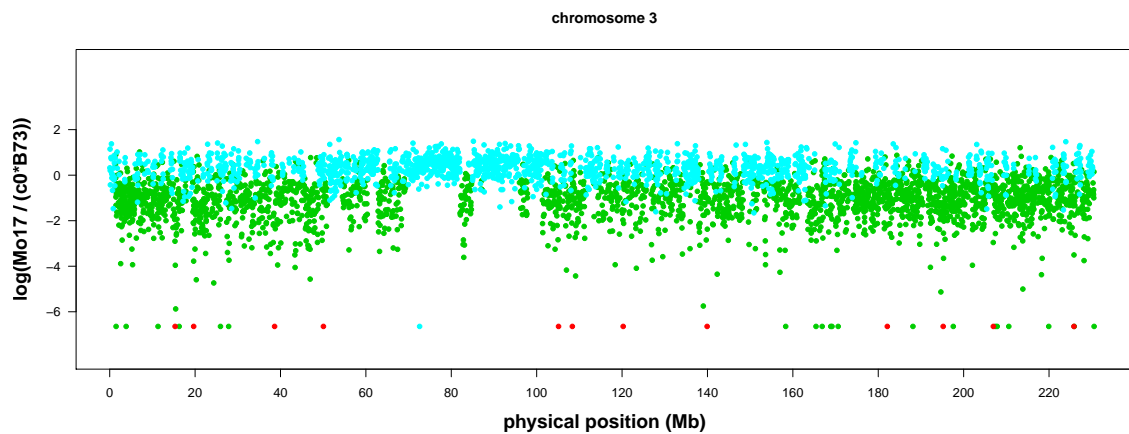

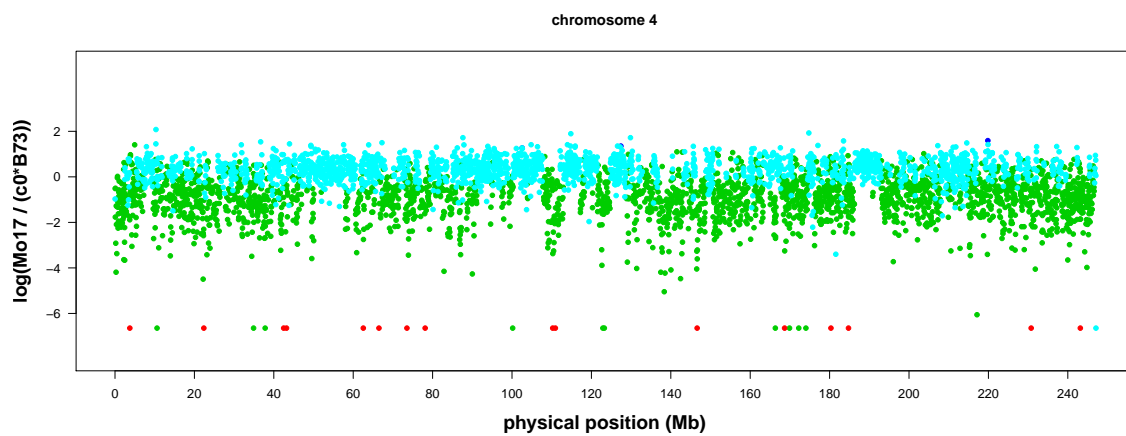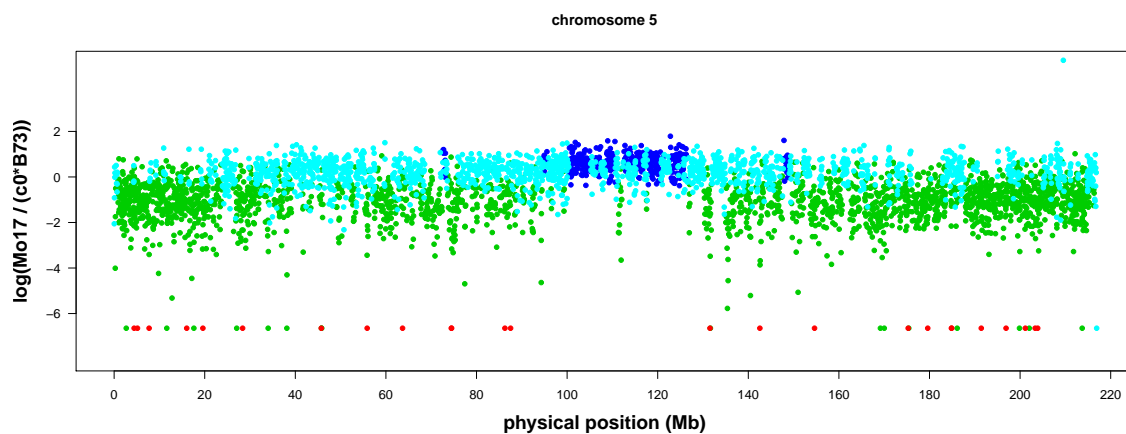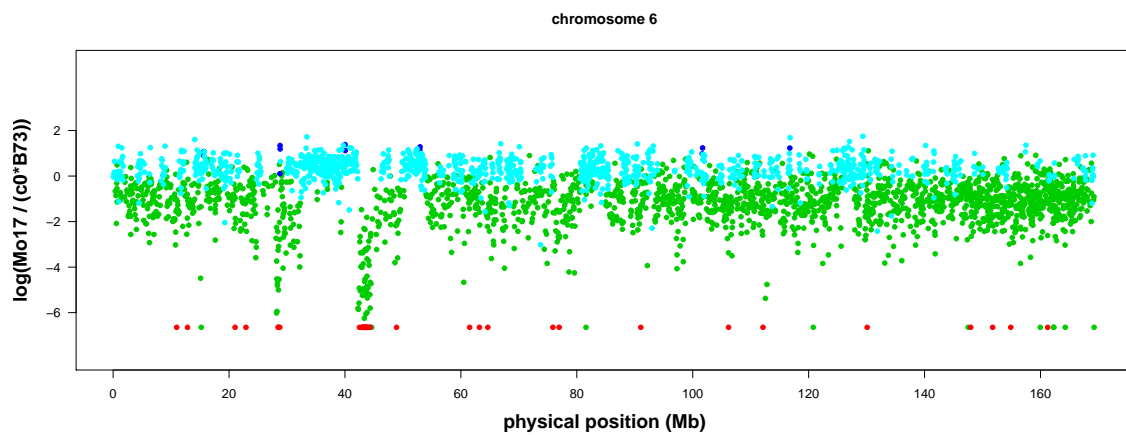

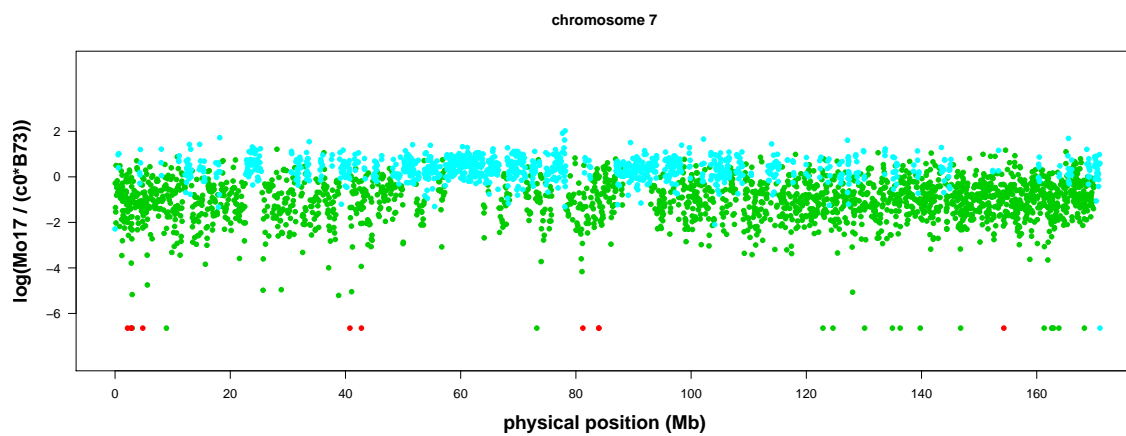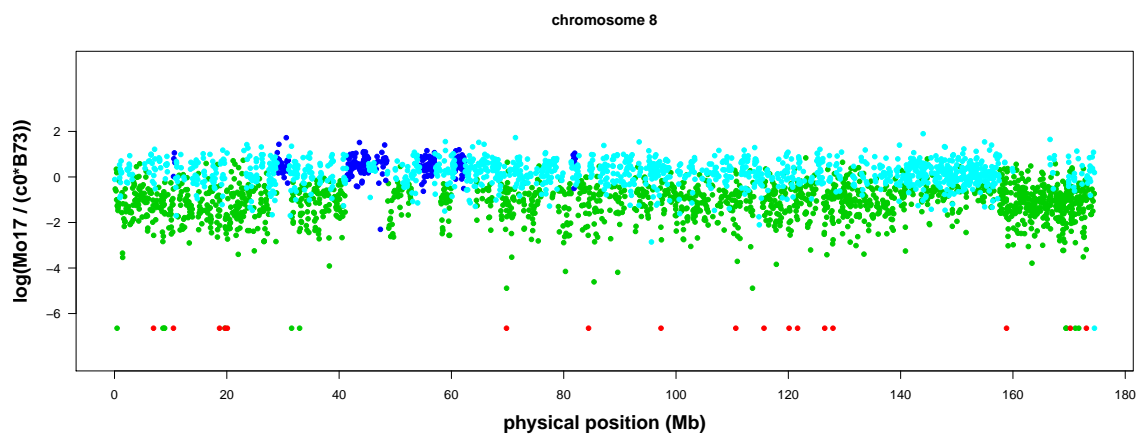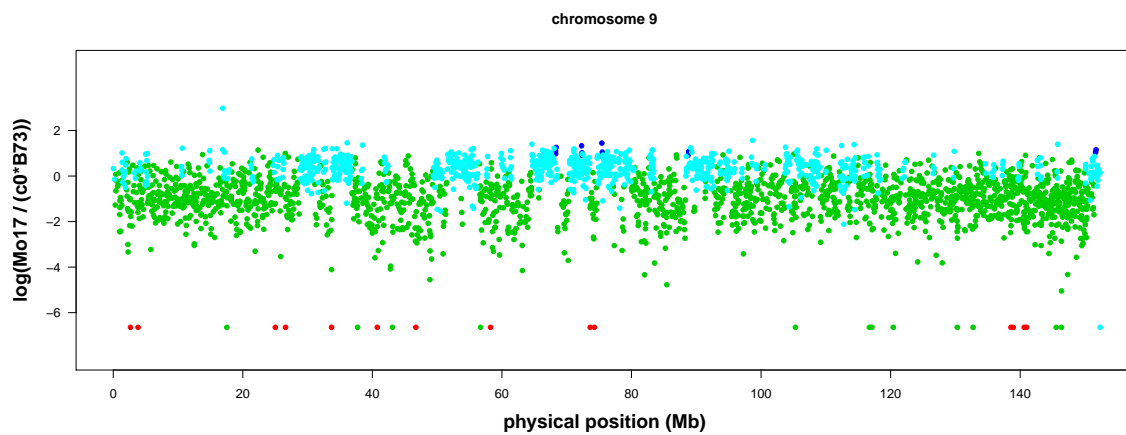

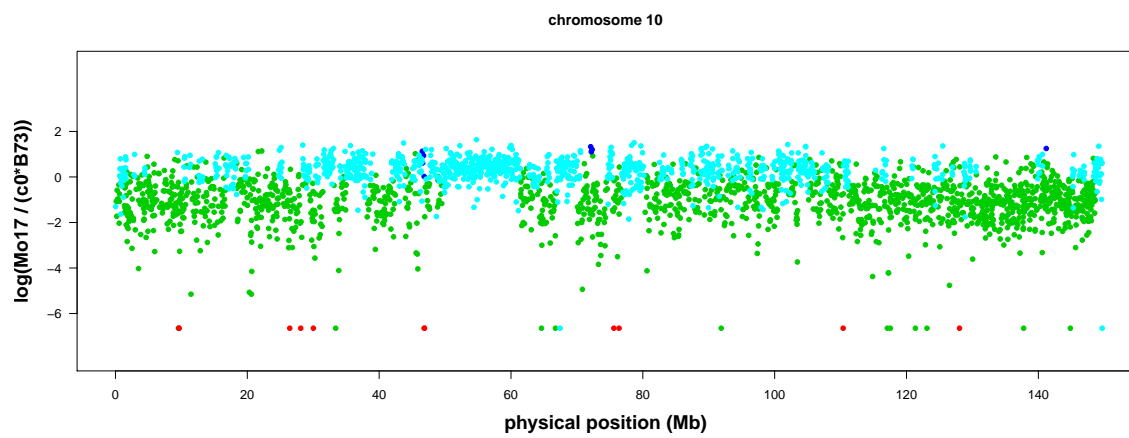

Supplement: Additional file 2 — Appendix 2 – Maize CNV Detection Results. [file 1471-2105-15-109-S2.pdf]
